# First report of furmonertinib as a first-line treatment in advanced lung adenocarcinoma patients harboring EGFR exon 20 insertion mutations after the kinase domain αC-helix: Two case reports and a literature review

**DOI:** 10.1097/MD.0000000000036667

**Published:** 2023-12-29

**Authors:** Huan Han, Xiao Zhang, Xiao Liu, Jiuzhou Zhao, Jianbo Zhang, Jianwei Zhang, Hui Zhu, Shuyue Jiao, Hong Tang

**Affiliations:** a Department of Medical Oncology, The Affiliated Cancer Hospital of Zhengzhou University and Henan Cancer Hospital, Zhengzhou, China; b Department of Radiotherapy, The Affiliated Cancer Hospital of Zhengzhou University and Henan Cancer Hospital, Zhengzhou, China; c Department of Pathology, The Affiliated Cancer Hospital of Zhengzhou University and Henan Cancer Hospital, Zhengzhou, China; d Department of Medical Iconography, The Affiliated Cancer Hospital of Zhengzhou University and Henan Cancer Hospital, Zhengzhou, China; e Department of Respiratory Medicine, Luohe Central Hospital, Luohe, China.

**Keywords:** EGFR ex20ins mutation, EGFR TKIs, first-line, furmonertinib, lung adenocarcinoma

## Abstract

**Rationale:**

Many studies have shown that first- and second-generation epidermal growth factor receptor tyrosine kinase inhibitors are less effective in patients with epidermal growth factor receptor (EGFR) exon 20 insertion (ex20ins) mutations. The efficacy of third-generation epidermal growth factor receptor tyrosine kinase inhibitors is still under investigation. Although new targeted tyrosine kinase inhibitors and monoclonal antibody-based agents have made significant advances in the treatment of epidermal growth factor receptor exon 20 insertion (EGFR ex20ins) mutation, the efficacy of these novel agents is not quite satisfactory. Platinum- and pemetrexed-based chemotherapy remains the standard first-line treatment for patients harboring EGFR ex20ins mutation.

**Patient concerns:**

We report for the first time 2 Chinese patients diagnosed with advanced lung adenocarcinoma with EGFR ex20ins mutations after analysis of the αC-helix sequence by next-generation sequencing. Both patients were treated with furmonertinib as the first-line therapy.

**Interventions:**

The first case included a 38-year-old female who had an EGFR ex20ins mutation (p.S768_D770dupSVD). After 1 month of treatment with furmonertinib, her symptoms of pain and cough were significantly alleviated. She achieved a partial response according to response evaluation criteria in solid tumors.^[1]^ The final progression-free survival was 8.13 months. The second case included a 40-year-old male who had an EGFR ex20ins mutation (p.N771_P772insVal). He had a good response to furmonertinib and exhibited stable disease according to response evaluation criteria in solid tumors with a progression-free survival of 10.90 months.

**Outcomes:**

Both patients experienced significant improvement in symptoms and prolonged survival after furmonertinib was used as first-line treatment. Side effects were limited but manageable.

**Conclusion:**

The present study indicates that furmonertinib may be a first-line treatment option for patients with non-small cell lung cancer harboring EGFR ex20ins mutation.

## 1. Introduction

Furmonertinib mesylate (AST2818) is a novel irreversible third-generation epidermal growth factor receptor tyrosine kinase inhibitor (EGFR TKI). It has been approved by the China National Medical Products Administration for the treatment of patients with locally advanced or metastatic non-small cell lung cancer (NSCLC) harboring sensitive EGFR mutations and the T790M resistance mutation.^[2,3]^ Furmonertinib has a broader safety window due to its unique trifluoroethoxypyridine structure. Both furmonertinib and its main metabolite, AST5902, have been shown to have high antitumor activity and high selectivity.^[4]^ However, the clinical efficacy of furmonertinib in patients with rare mutations, such as epidermal growth factor receptor exon 20 insertion (EGFR ex20ins) mutation, L861Q, G719X and S768I, has not been adequately characterized. Here, we present 2 patients with advanced lung adenocarcinoma harboring EGFR ex20ins mutation who responded well to first-line furmonertinib, achieving progression-free survival (PFS) of 8.13 months and 10.90 months, respectively.

## 2. Case presentation

The study was approved by the Ethics Committee of Henan Cancer Hospital. Informed consent was obtained from both patients.

### 2.1. Case 1

**Figure 1.**
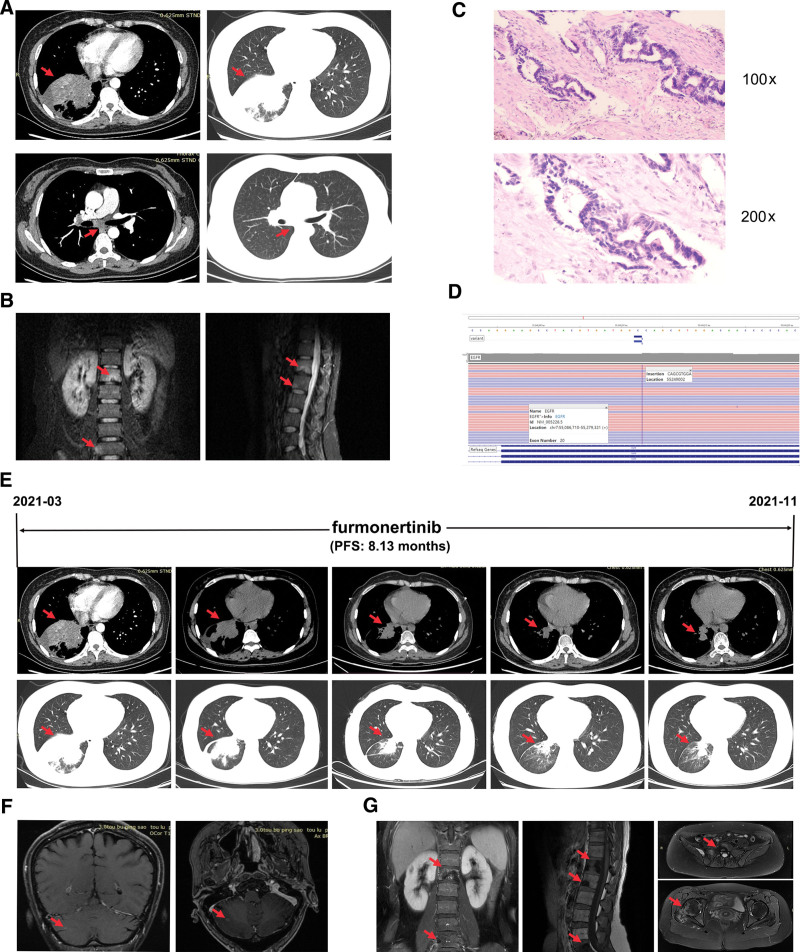
Baseline date of case one patient and dynamic changes in the tumor during treatment. (A) Computed tomography (CT) images. CT scans showed a 41*47 mm density mass in the lower right hilar with obstructive atelectasis. (B) Magnetic resonance imaging (MRI). MRI showed multiple bone metastases. (C) Hematoxylin-eosin (H&E) staining suggested lung adenocarcinoma. (D) Next-generation sequencing showed a p.S768_D770dupSVD (c.2300_2301ins CAGCGTGGA) mutation in EGFR ex20. (E) Representative computed tomography images at various points. CT scans revealed lesion in the lower right lung. (F) Magnetic resonance imaging suggested cerebral metastasis (G) and a larger extent of bone metastases. The timeline of therapy, therapeutic regimens and tumor progression are indicated (top). The lesions are indicated by red arrows. PFS = progression-free survival.

A 38-year-old nonsmoking woman was admitted to our hospital in March 2021 with a history of back pain and cough. A computed tomography (CT) scan revealed a 41*47 mm dense mass in the right lower lobe with obstructive atelectasis, mediastinal lymph node enlargement, and no clear local lesions in the brain (Fig. 1A). Magnetic resonance imaging (MRI) showed multiple bone metastases (Fig. 1B). Lung fine-needle aspiration biopsy revealed pulmonary adenocarcinoma (Fig. 1C). Her tumor DNA extracted from the tissue was subjected to DNA sequencing analysis by next-generation sequencing. Genetic testing revealed that the patient had an EGFR ex20ins mutation (c.2300_2301ins CAGCGTGGA, p.S768_D770dupSVD, reference sequence: NM_005228). The mutation abundance was 73.35% (Fig. 1D). Based on these data, the patient was diagnosed with stage IVB (cT2bN3M1c) lung adenocarcinoma.

Given the patient’s physical condition, her family declined chemotherapy. Therefore, the patient received first-line furmonertinib (240 mg/day) in March 2021. The concomitant treatment plan consisted of zoledronic acid injection for bone metastases. One month posttreatment (April 2021), the patient’s symptoms of pain and cough were significantly alleviated. Her quality of life was greatly improved. As shown in Figure 1E, the size of the tumor lesion in the right lower lung was significantly reduced (size: 33*24 mm), and a partial response (PR) was achieved according to Response Evaluation Criteria in Solid Tumors (RECIST) 1.1.^[1]^ Unfortunately, the patient presented with a grade 2 rash. After topical treatments based on hydrocortisone 2.5% and doxycycline 100 mg once daily, the rash severity was decreased from grade 2 to grade 1. In the meantime, furmonertinib was administered at the same dose. After 1 month, the grade 2 rash appeared again. Therefore, as required by the protocol, in June 2021, a dose reduction to 160 mg/day was performed. Subsequently, the skin toxicity improved significantly, and the patient tolerated treatment better without further dose reduction or drug interruption. In June and August 2021, a reduction in the right lung lesion size of 42% and 53%, respectively, was recorded according to response evaluation criteria in solid tumors (RECIST 1.1) (Fig. 1E). The patient remained on treatment with furmonertinib and retained her PR status with a PFS of 8.13 months.

At the end of November 2021, the patient complained of severe pain in the right lower extremity and back, which even interfered with walking. CT scans revealed an increased tumor lesion in the right lung (Fig. 1E). MRI showed a metastatic lesion in the brain and an increased extent of bone metastases (Fig. 1F and G). The patient was evaluated as having progressive disease according to RECIST 1.1. Due to economic difficulties, she refused to undergo additional tissue biopsy or liquid biopsy to evaluate circulating tumor DNA. The patient was then treated with albumin-paclitaxel and cisplatin plus bevacizumab as second-line therapy. However, after 4 cycles of treatment, the disease continued to progress. She was then switched to daily oral anlotinib as third-line treatment. The patient did not have regular clinic visits for financial reasons, and she died in December 2022.

### 2.2. Case 2

**Figure 2.**
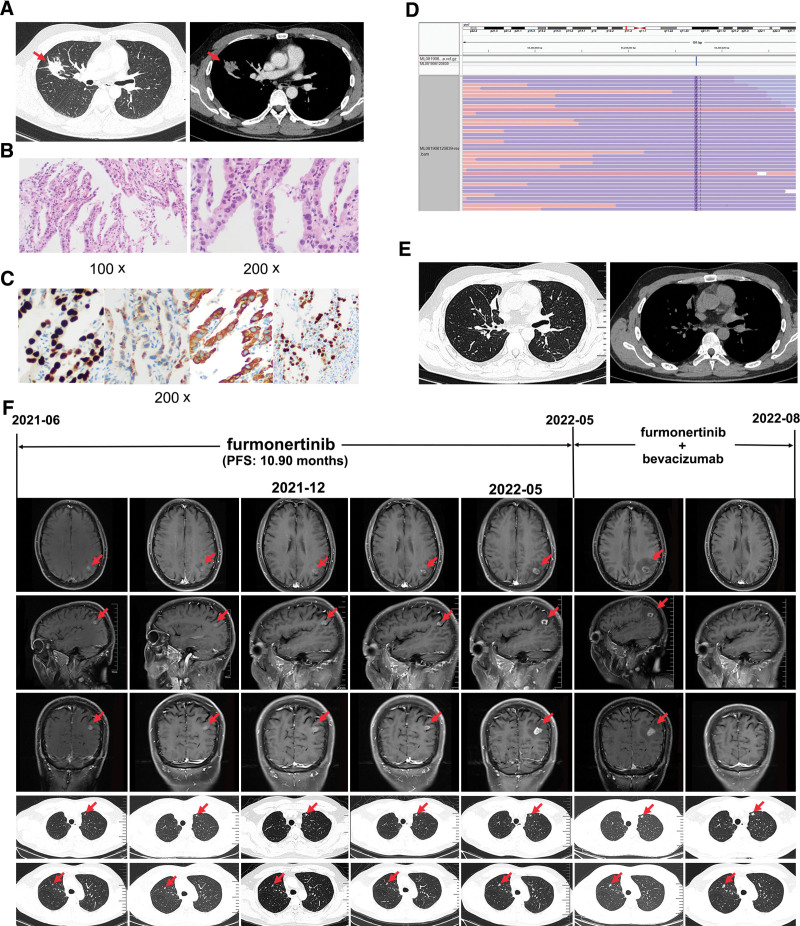
Baseline date of case two patient and dynamic changes in the tumor during treatment. (A) Computed tomography (CT) images before surgery. CT scan showed a 22*25 mm mass in the right upper lobe. (B) Hematoxylin-eosin (H&E) staining suggested lung adenocarcinoma (x100 and x200 respectively). (C) Immunohistochemistry (IHC) staining: TTF-1, Napsin A, CK7, Ki-67 was positive respectively (x200). (D) Next-generation sequencing showed a p.N771_P772insVal (c.2312_2313insTGT) mutation in EGFR ex20. (E) Computed tomography (CT) images after surgery. CT scans showed that the tumor was completely removed. (F) Magnetic resonance images and computed tomography images at various points. MRI revealed cerebral metastasis. CT scans revealed multiple small nodules in bilateral lungs. The timeline of therapies, therapeutic regimens and tumor progression are indicated (top). The lesions are indicated by red arrows. MRI = magnetic resonance imaging, PFS = progression-free survival.

A 40-year-old man who was a nonsmoker presented to our hospital in May 2019 for pulmonary nodules that were detected during a physical examination. The patient underwent a comprehensive CT scan, which confirmed a 22*25 mm mass in the right upper lobe (Fig. 2A). Brain MRI and bone imaging revealed no metastases. CT-guided core needle biopsy of the lung and immunohistochemistry revealed pulmonary adenocarcinoma (Fig. 2B and C). Genotyping of lung cancer-related genes by next-generation sequencing using the patient’s pathological tissue sample suggested a rare EGFR ex20ins mutation (c.2312_2313insTGT, p.N771_P772insVal, reference sequence: NM_005228.3). The mutation abundance was 22.19% (Fig. 2D). Based on these data, the patient was diagnosed with stage IA (cT1N0M0) lung adenocarcinoma.

The patient underwent right lobectomy and lymphadenectomy for stage IA (pT1N0M0) lung adenocarcinoma (Fig. 2E). He was then discharged after surgery and followed up regularly. There was no recurrence for 2 years. Unfortunately, in June 2021, MRI showed brain metastasis (size: 10*8*6 mm) (Fig. 2F). In addition, multiple small nodules appeared in the bilateral lungs (Fig. 2F). The patient refused platinum-containing chemotherapy, the standard of care for his condition. Given that the difficult in passing the blood–brain barrier results in lower drug concentrations in the CSF than in the peripheral blood, the patient was treated with furmonertinib (240 mg/day) as the first-line therapy. After 1 month of treatment (July 2021), MRI showed that the enhanced nodules in the left parietal lobe became blurred and slightly smaller (size: 10*6*6 mm) (Fig. 2F). After 6 months of treatment (December 2021), the primary brain metastasis lesion was observed to be slightly enlarged on brain MRI (size: 11*10*9 mm) (Fig. 2F). The sizes of multiple small nodules in both lungs remained stable (Fig. 2F). The patient exhibited stable disease (SD) according to RECIST 1.1. He was subsequently treated with stereotactic radiosurgery for the brain lesion (18 Grays in 1 fraction) on the advice of a consulting radiologist. The patient remained on treatment with furmonertinib (240 mg/day) and achieved SD with a PFS of 10.90 months.

In May 2022, a brain MRI revealed that the lesion continued to increase in size (size: 17*12*15 mm), and CT scans showed that the size of multiple small nodules in the bilateral lungs also increased (Fig. 2F). The patient was characterized as having progressive disease according to RECIST 1.1. Tissue biopsy could not be performed due to the infeasibility of obtaining additional tissue, and the patient refused to undergo liquid biopsy to assess circulating tumor DNA. Therefore, with the patient’s consent, reirradiation was performed with CyberKnife Stereotactic Radiotherapy (21 Gy in 3 fractions), and bevacizumab was added to furmonertinib (240 mg/day) beginning in June 2022. Surprisingly, the cranial MRI 2 month later revealed that the intracranial lesion had almost disappeared. Chest CT confirmed that both nodules were stable. According to RECIST 1.1, the patient was evaluated as having PR, and he continued to receive furmonertinib treatment. Due to COVID-19, the patient did not receive regular treatment but continued to take oral furmonertinib (240 mg/day). Unfortunately, the disease reappeared again in February 2023. Subsequently, the patient was treated with platinum-containing chemotherapy regimens. Although he received brain radiotherapy, we believe that the targeted drug furmonertinib played a key role. Throughout treatment with furmonertinib, no grade 3 or higher adverse events (AEs) occurred. The treatment-related AEs associated with furmonertinib were grade 1 or 2 diarrhea and paronychia, and there was no treatment interruption or dose reduction. These AEs occurred during the first month of furmonertinib therapy and were relieved with corresponding symptomatic therapies.

## 3. Discussion

Here, we report for the first time the clinical efficacy of first-line furmonertinib in patients with advanced NSCLC harboring uncommon EGFR ex20ins mutations (p.S768_D770dupSVD and p.N771_P772insVal) in the real-world. The patients benefited from furmonertinib, with PFS times of 8.13 months and 10.90 months, respectively.

EGFR mutations are major driver mutations in NSCLC. In particular, EGFR-activating mutations have resulted in routine use of epidermal growth factor receptor tyrosine kinase inhibitors (EGFR TKIs). However, NSCLC patients with EGFR ex20ins mutations have reduced response to EGFR TKIs compared to those harboring classical mutations (EGFR ex19Del and EGFR ex21L858R).^[5]^ Currently, no EGFR TKIs are approved for first-line treatment of NSCLC patients with EGFR ex20ins mutations in China. The standard of care is platinum-based chemotherapy, with a median progression-free survival (mPFS) of 4.1 to 6.4 months and a response rate of 50% to 63%.^[5–7]^ There is a significant clinical need to improve the outcome of NSCLC patients harboring EGFR ex20ins mutations.

Ex20ins mutations account for 4% to 10% of all EGFR mutations and are the third most common mutation type in NSCLC.^[8]^ EGFR ex20ins mutation contains all the amino acids (762–823) of other exon 20 mutations, except for the classic resistance mutation T790M.^[8]^ Most EGFR ex20ins mutations are in the loop following the αC-helix of the EGFR kinase domain (M766-C775), and a few occur in the αC-helix (E762-Y764).^[9]^ Many studies have showed that EGFR ex20ins mutations occurring in the C-terminal loop of the αC-helix can be subdivided into 2 subgroups: near-loop (A767-P772) and far-loop (H773-C775) ex20ins mutations.^[8,9]^ The near-loop region is the region most frequently affected by EGFR ex20ins mutation, and nearly 70% of EGFR ex20ins mutations are in this region.^[9]^ Notably, both mutation types (p.S768_D770dupSVD and p.N771_P772insVal) we reported were in the near-loop region.

Studies have shown that the response of EGFR ex20ins mutations to EGFR TKIs is heterogeneous. It was reported that insertions in the αC-helix (for example, EGFR A763-Y764insFQEA) were associated with sensitivity to available EGFR TKIs.^[10]^ In contrast, those in the loop following the αC-helix promoted the activation of the EGFR kinase domain without significantly diminishing ATP affinity or increasing the affinity for EGFR TKIs, resulting in resistance to first- and second-generation EGFR TKIs.^[10]^ Recent studies have suggested that first- and second-generation EGFR TKIs are less effective in patients with the EGFR ex20ins mutation (mPFS: 2.0–2.7 months; ORR: 0%–8.7%).^[11–13]^ Nevertheless, several studies have shown durable responses to afatinib in patients who harbor exon20ins mutations near codon 770.^[14–16]^ Similarly, the efficacy of third-generation EGFR TKIs is also controversial.

Floc’h et al^[17]^ reported that osimertinib and AZ5104 (a circulating metabolite of osimertinib) inhibited the signaling and growth of EGFR ex20ins mutant cell lines in vitro and had a long-lasting antitumor growth effect. In a phase I/II study, osimertinib (80 mg/day) exhibited limited clinical activity in patients harboring EGFR ex20ins mutation (mPFS: 3.8 months; mOS: 15.8 months; ORR: 0%).^[18]^ In 2 other prospective studies,^[19,20]^ the use of osimertinib (160 mg/day) as first- and later-line treatment in NSCLC patients harboring the EGFR ex20ins mutation showed moderate efficacy (mPFS: 6.8–9.7 months; ORR: 24%–28%). However, it was associated with a high incidence of AEs. The most common AEs were diarrhea (72%–76%), fatigue (44%–67%) and decreased platelet count (20%–67%).

Both furmonertinib and its main metabolite (AST5902) can effectively treat cancers with EGFR sensitive mutations and T790M drug-resistant mutations. They are highly selective and have almost no inhibitory effect on wild-type cells.^[3]^ The FURLONG study reported that furmonertinib achieved a significantly longer mPFS than gefitinib as first-line therapy in Chinese patients with EGFR mutation-positive advanced NSCLC (mPFS: 20.8 months vs 11.1 months; HR 0.44, 95% CI 0.34–0.58; *P* < .0001).^[3]^ This study also showed that first-line furmonertinib had superior efficacy to gefitinib in patients with EGFR sensitive mutation-positive advanced NSCLC with central nervous system (CNS) metastases (mPFS: 20.8 months vs 9.8 months; HR 0.40, 95% CI: 0.23–0.71; *P* = .0011).^[21]^ Not surprisingly, the PFS with furmonertinib was superior to that with gefitinib, regardless of the presence of CNS metastases.

Similar to these classic EGFR mutations, CNS metastases are another major challenge for NSCLC patients with EGFR ex20ins mutations and are associated with poor prognosis. While 2 novel EGFR ex20ins drugs have recently been approved by the FDA (amivantamab and mobocertinib), neither has demonstrated CNS activity.^[22,23]^ Studies have shown that third-generation EGFR TKIs exhibit excellent CNS activity because of their ability to penetrate the blood–brain barrier and ultimately be distributed to brain tissue.^[24]^ The osimertinib and almonertinib drug prototypes can be distributed to brain tissue, while both the furmonertinib drug prototype and its major metabolites can be distributed to brain tissue.^[21,24,25]^ The multicenter phase 2 POSITION20 trial showed a reduction in brain lesion size in all patients with CNS disease, suggesting a benefit of osimertinib in this subgroup.^[19]^ In addition, numerous single-institution studies are currently evaluating 160 mg daily furmonertinib in patients with EGFR ex20ins mutation and CNS metastases (NCT05465343; NCT05379803).

Furthermore, several ongoing clinical studies have reported that furmonertinib showed good efficacy and good tolerability in NSCLC patients harboring EGFR ex20ins mutation. The FAVOUR 1 study (phase Ib study, NCT04858958) reported that furmonertinib effectively inhibited Ba/F3 cells expressing EGFR ex20ins with a median IC50 of 11 to 20 nm.^[26]^ The preliminary result of the FAVOUR 1 study showed that advanced NSCLC patients harboring EGFR ex20ins mutation who were treated with furmonertinib (240 mg/day) showed tumor shrinkage in the target lesion (median best percent change, −43.0% [−72.3%, −3.0%]). The most common adverse reactions were diarrhea, paronychia and skin cracks (30% each), and no grade 3 or higher AEs were observed.^[26]^ A multicenter phase Ib clinical study (NCT04958967) is evaluating the efficacy and safety of furmonertinib at different doses (160 mg/day and 240 mg/day) in patients with locally advanced or metastatic NSCLC harboring EGFR ex20ins mutation.^[27]^ Of note, there are also 2 ongoing clinical trials (EXCLAIM-2 and PAPILLON) for first-line treatment of patients with locally advanced or metastatic NSCLC harboring EGFR ex20ins mutation.

Furmonertinib has also recently been used in a real-world setting. At the 2022 American Society of Clinical Oncology meeting, 15 patients with EGFR ex20ins mutation received furmonertinib (160 mg/mg) as ≥ 2nd line treatment, of which 8 patients had PR and 7 patients had SD. No grade 3 or higher AEs occurred.^[28]^ This result further confirmed the efficacy and safety of furmonertinib. Our team previously reported that a patient with a rare EGFR ex20ins N771_P772insH mutation was treated with furmonertinib (160 mg/day) as second-line treatment. The treatment had very good efficacy and high tolerability, with the patient achieving a PFS of 10.0 months, a DOR of 8.0 months and an OS of 22.0 months (not yet achieved).^[29]^ In addition, 2 other case reports also confirmed the efficacy and safety of high-dose furmonertinib.^[30,31]^ Although these cases reported on patients harboring EGFR ex20ins mutation who were treated with furmonertinib, all of them received the treatment as second-line or multiline therapy. We reported for the first time 2 cases of patients harboring EGFR ex20ins mutation who received first-line furmonertinib treatment, resulting in very good clinical efficacy and acceptable safety. However, large prospective studies are needed to validate this result.

Notably, these studies have shown that different EGFR TKIs have different efficacies in patients with EGFR ex20ins mutations, and to further explore the reasons for this difference, we need to have a good understanding of the drug structure and the effect on drug binding. Osimertinib has a large terminal 1-methylindole group attached directly to the rigid pyrimidine core. This large inflexible group reduces the ability of osimertinib to reach Cys797 residues in ex20ins mutant EGFR.^[32]^ Poziotinib is centered on a less rigid quinazoline core with small terminal substituents that make it easier to bind to the drug binding pocket of ex20ins mutant EGFR.^[32]^ The design of the isopropyl ester structure of mobocertinib leads to an increased affinity for ex20ins mutant EGFR.^[33]^ Based on the retention of the unsaturated acrylamide bond and pyrimidine ring, furmonertinib has a unique trifluoroethoxypyridine structure (highly hydrophobic). Similar to osimertinib, furmonertinib is designed to irreversibly bind Cys797 in EGFR.^[34]^ The hollow hydrophobic pocket composed of the hydrophobic amino acids L792 and M793 in the ATP-binding region of EGFR has high affinity, which improves the activity of the drug itself. Furmonertinib also has different metabolic properties that reduce the production of nonselective metabolites.^[4,34]^ In addition, we used 7LGS and 4LRM protein crystal structures as receptor protein objects and calculated the binding free energy of different TKIs for EGFR ex20ins mutations by kinetic methods. The results showed that both furmonertinib and osimertinib had good binding power, with furmonertinib having the most significant binding.

In recent years, many clinical trials have also reported the efficacy of several novel EGFR TKIs and anti-EGFR monoclonal antibodies in NSCLC patients harboring EGFR ex20ins mutation, as shown in Table 1. The emergence of these novel drugs has clearly provided more treatment options for patients with advanced NSCLC with EGFR ex20ins mutation, which may benefit more patients. Notably, these studies did not distinguish between different EGFR ex20ins mutations. The position, size, and subtype of the amino acid inserted in the EGFR ex20ins mutation may underly the heterogeneous response to TKIs seen in the clinic. Afatinib achieved durable responses in a small number of NSCLC patients with specific EGFR ex20ins mutations.^[14–16]^ Both Fang et al^[36]^ and Qin et al^[35]^ reported that osimertinib could effectively inhibit specific subtypes of EGFR ex20ins mutations. However, the first-line application of osimertinib showed limited effects, especially for patients with p.S768_D770dup and p.A767_V769dup, implying poor outcomes of its clinical application.^[35]^ Preclinical test results showed that furmonertinib could effectively inhibit a variety of ex20ins mutant EGFR subtypes, including p.H773_V774insNPH, p.V769_D770insASV, p.D770_N771insSVD and p.D770_N771NPG. Remarkably, in our case, the EGFR ex20ins mutation was located behind the αC-helix (p.S768_D770dupSVD); first-line treatment with third-generation furmonertinib showed good affinity, and the patient showed a good clinical response. Incidentally, the rare EGFR ex20ins mutation p.N771_P772insVal in the present case has not been previously described. Considering such surprising results, the p.N771_P772insVal mutation may be a specific subtype of EGFR ex20ins mutation that indicates sensitivity to furmonertinib. Furmonertinib may be a promising therapy for patients with this subtype of EGFR ex20ins mutation. Given the heterogeneity of EGFR ex20ins mutations, it is important to identify the EGFR subtypes that are associated with response to EGFR TKIs.

**Table 1:** Clinical trial summary about EGFR 20ins.

Treatment	Mechanism of action	N	ORR	mPFS		Primary endpoint reached? (Y/N)	Level of evidence	Clinical trial
Poziotinib	EGFR TKI	90	27.8%	5.5	Y		Phase 2 clinical trial	NCT03318939 (ZENITH20)
Mobocertinib	EGFR TKI	96	28%	7.3	Y		Phase 1/2 clinical trial	NCT02716116 (EXCLAIM) NCT04129502 (EXCLAIM-2)
Osimertinib	EFRG TKI	20	25%	9.7	Y		Phase 2 clinical trial	NCT03414814 NCT03191149 (EA5162)
CLN-081	EGFR TKI	-	-				Phase 1/2 clinical trial	NCT04036682
luminespib	Hsp90 inhibitor	29	17%	2.9	Y		phase 2 clinical trial	NCT01854034
Amivantamab	EGFR-Met bispecific antibody	81	40%	8.3	Y		phase 1 clinical trial	NCT02609776 (CHRYSALIS)
Furmonertinib	EGFR TKI	30	60%	-	-		Phase 1b clinical trial	NCT05364073 NCT04958967
Afatinib + Cetuximab	EGFR TKI + EGFR antibody	17	47%	5.5	N		Phase 2 clinical trial	NCT03727724
Sunvozertinib (DZD9008)	EGFR TKI	79	58.9%	-	N		Phase 1/2 clinical trial	NCT03974022 (WK-KONG1) CTR20192097 (WU-KONG2) CTR20211009 (WU-KONG6)
BLU-451	EGFR TKI	-	-	-	-		Phase 1/2 clinical trial	NCT05241873
Osimertinib + Bevacizumab	EGFR TKI + EGFR antibody	-	-	-	-		-	NCT04974879

* FDA approval granted.

** Data collected on November 01, 2022.

*** ORR, Objective Response Rate; mPFS: median progression-free survival.

Based on the above studies, we found that high-dose third-generation EGFR TKIs are more effective for patients with EGFR ex20ins mutation, but the consequence of high-dose EGFR TKI treatment is more severe side effects. As a novel third-generation EGFR TKI, high-dose furmonertinib not only showed encouraging antitumor activity but also had a good safety profile. Furmonertinib may be a therapeutic option for advanced NSCLC patients harboring EGFR ex20ins mutation.

However, the cases also had some limitations, such as refusal of treatment for economic and personal reasons and the small case size. Considering the nature of the case reports, some important issues still need to be investigated. Our team will continue to expand the sample size to determine the clinical efficacy of furmonertinib as first-line therapy in patients with locally advanced or metastatic NSCLC harboring EGFR ex20ins mutation. The safety and efficacy of 160 mg/day and 240 mg/day furmonertinib will be compared to find the most appropriate therapeutic dose. We will also explore the potential mechanism by which furmonertinib has effects against NSCLC with EGFR ex20ins mutation and compare the differences between furmonertinib and other EGFR TKIs.

## 4. Conclusion

Overall, we first reported the cases of 2 lung adenocarcinoma patients harboring EGFR ex20ins mutations (p.S768_D770dupSVD and p.N771_P772insVal) who received furmonertinib as first-line treatment and achieved PFS times of 8.13 and 10.90 months, respectively. The success of furmonertinib treatment may provide a novel option for patients with these EGFR ex20ins mutations. However, there are various types of EGFR ex20ins mutations, and the efficacy of targeted therapies may vary depending on the type of mutation; thus, further research is needed.

## Acknowledgements

We thank two patients and their families for participating in this study. The patient involved in two case reports gave them informed consent authorizing use and disclosure of their health information.

## Author contribution

**Conceptualization:** Huan Han, Xiao Zhang, Xiao Liu, Hong Tang.

**Data curation:** Huan Han, Xiao Zhang, Xiao Liu, Jiuzhou Zhao, Jianbo Zhang, Jianwei Zhang, Shuyue Jiao, Hong Tang.

**Formal analysis:** Huan Han, Jianwei Zhang.

**Funding acquisition:** Hong Tang.

**Investigation:** Xiao Zhang, Hui Zhu, Shuyue Jiao.

**Methodology:** Huan Han, Xiao Liu, Jiuzhou Zhao, Jianbo Zhang, Hong Tang.

**Resources:** Jianbo Zhang.

**Supervision:** Jianwei Zhang, Hui Zhu, Hong Tang.

**Visualization:** Xiao Liu.

**Validation:** Jiuzhou Zhao, Hui Zhu.

**Writing – original draft:** Huan Han.

**Writing – review & editing:** Huan Han, Xiao Zhang.
